# Advanced 2D XRF imaging of uranium oxidation states using HERFD at the U M_4_ edge†

**DOI:** 10.1039/d4cc06211f

**Published:** 2025-02-05

**Authors:** Elena F. Bazarkina, Kimberly V. Lau, Anthony Chappaz, Evgeny Bastrakov, Barbara Etschmann, Joël Brugger, Madeline Marshall, Frances M. Meyer, Christopher J. Boreham, Lucia Amidani, Kristina O. Kvashnina

**Affiliations:** a Institute of Resource Ecology, Helmholtz Zentrum Dresden-Rossendorf (HZDR) PO Box 510119 01314 Dresden Germany kristina.kvashnina@esrf.fr; b The Rossendorf Beamline at ESRF, The European Synchrotron CS40220 38043 Grenoble Cedex 9 France; c Dept of Geosciences and the Earth and Environmental Systems Institute, Penn State University University Park PA 16802 USA; d STARLAB, Department of Earth and Atmospheric Sciences, Central Michigan University 17 MI 48859 USA; e Geoscience Australia GPO Box 378 Canberra ACT 2601 Australia; f School of Earth Atmosphere and Environment, Monash University Clayton VIC 3800 Australia; g Department of Earth and Environment, Albion College Albion MI 49224 USA

## Abstract

Uranium is found in various types of rocks. HERFD-XRF imaging at the U M_4_ edge is a novel non-destructive technique that visualizes the distribution of U(iv), (v), and (vi) oxidation states at concentration levels ranging from ppm to wt%, offering unprecedented insights into uranium habitat and valence.

## Abstract 2

Identification of U(iv), U(v) and U(vi) oxidation states in rocks can be achieved with HERFD-XRF imaging at the uranium M_4_ edge.

## Graphical abstract

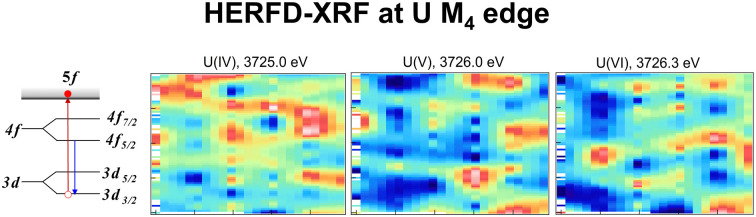

Understanding uranium (U) geochemistry is essential for developing sustainable solutions for nuclear waste management and environmental remediation.^1,2^ Uranium exists in several oxidation states in geochemical systems; U(iv)-bearing minerals are insoluble, whereas some U(vi) phases are highly soluble and hence pose a great risk of groundwater contamination.^3^ The intermediate U(v) oxidation state has been thought to be rare but can be still been found in stable mineral phases.^4–7^ The presence of multiple oxidation states complicates the behaviour of U in natural systems. By studying how U oxidation states are distributed within Earth materials, strategies can be designed to immobilize U, preventing its migration in the environment and contributing to cleaner, safer energy production and waste containment, aligning with global sustainability goals.

Uranium can be concentrated by various geological processes, forming ore deposits at the wt% level, or dispersed in the environment down to ppm levels or lower. Uranium at ppm levels can be found in natural waters, sediments, soils, and many types of rocks, with sedimentary rocks (*e.g.*, shales and phosphorites) containing relatively high concentrations of U. The physical state of uranium and its speciation can also vary significantly. It can be found incorporated into mineral structures, sorbed onto surfaces, integrated into organic matter, retained within soils, or present as colloids, amorphous phases, and nanoparticles.^1^ A synchrotron-based, element-selective X-ray fluorescence (XRF) method is well-suited for detecting different chemical species and imaging the distribution of chemical elements.^8–17^ Oxidation states can be explored by XRF mapping at characteristic energies; these energies can be determined from features in X-ray absorption near edge structure (XANES) spectra using the total fluorescence yield (TFY)^18–22^ or high-energy resolution fluorescence detection (HERFD) modes. The advantages of HERFD are the ability to extract weak signals from a matrix exhibiting a high background and the improved energy resolution of the XANES spectra.^23,24^ HERFD-XANES measurements can be applied at the U L_3_ edge (∼17 166.0 eV) using the *L*α_1_ emission line (∼13 614.0 eV, 2p_3/2_–3d_5/2_ transitions).^25,26^ However, the oxidation state of U can be better probed by HERFD-XANES at the U M_4_ edge (∼3727.0 eV) using the Mβ emission line (∼3339.8 eV, 3d_3/2_–4f_5/2_ transitions, *cf.*Fig. 1a).^25,27^ Uranium has 7s^2^6d^1^5f^3^ electronic ground state configuration, with the number of 5f^*n*^ electrons varying with the oxidation state (*i.e.*, *n* = 2, 1 and 0 for U(iv), (v) and (vi), receptively). Thus, HERFD-XANES at the U M_4_ edge provides an exceptional opportunity to detect complex mixtures of U(iv), U(v) and U(vi) (see Fig. 1b and ref. 24, 25, 27–32). One of the key advantages of this technique is the sensitivity to U(v) detection and quantification which is difficult to achieve by other methods.

**Fig. 1:**
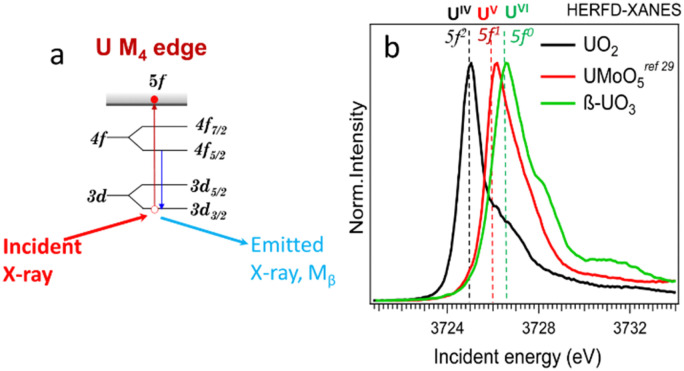
(a) Scheme of the absorption and emission processes at U M_4_ edge, (b) HERFD-XANES spectra of U(iv), (v), and (vi) reference compounds.

In this study, we combined the advantages of XRF and HERFD methods to create a new approach for the field of actinide science. The measurements were done using a Johann-type X-ray emission spectrometer^33^ on the BM20 (Rossendorf) beamline^34^ of the European Synchrotron Radiation Facility (ESRF) (additional information about the experiment and the assessment of potential X-ray-induced radiation damage is included in the ESI,†*cf*. Fig. SI1). The overall objective was to explore U M_4_ edge HERFD measurements for visualization of the 2-dimensional (2D) distribution of U oxidation states on natural samples with whole-rock U concentrations of ppm levels. Two types of sedimentary rocks were selected: (1) a phosphorite grainstone (USA) containing 80 ppm of U and (2) organic-rich carbonaceous shales (“Springvale” containing 50 ppm of U and coquinite “Westmoreland” containing 300 ppm of U, both from from the Toolebuc Formation of the Eromanga Basin, Australia^35^). All samples were well characterized previously (ESI†).

The principle of the new methodology is described below. First, HERFD-XANES of pure U(iv), U(v) and U(vi) reference compounds (Fig. 1) are measured to determine the energy shift between different oxidation states. There is a ∼1 eV shift between U(iv) and U(v) and ∼0.6 eV between U(v) and U(vi) compounds, shown in Fig. 1. Then, the HERFD-XRF maps are generated by scanning an area of the sample with the incident X-ray energy fixed at the maximum of the U(iv), U(v) and U(vi) HERFD-XANES white lines (see Fig. 1), resulting in a total of three energy maps. All U M_4_ edge HERFD-XRF maps (Fig. 2 and 3) show fluorescence intensity (in arbitrary units), which depends not only on the oxidation state but also on the total U concentration. Therefore, the relative differences between the three maps, recorded at 3725.0 (U(iv) max), 3726.0 (U(v) max) and 3726.6 (U(vi) max) eV, indicate the relationships between oxidation states and total U distribution.

**Fig. 2:**
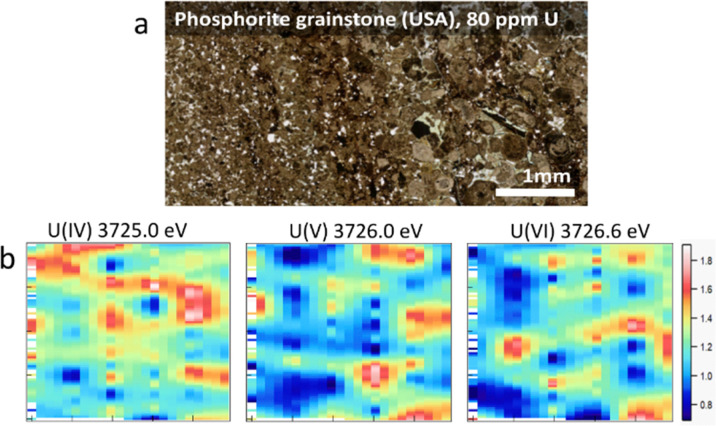
(a) Photo of a thin section of phosphorite grainstone (USA) containing 80 ppm of U, (b) HERFD-XRF maps showing the distribution of U(iv), U(v), and U(vi) in the selected area of 3.0 mm (vertical) × 5.0 mm (horizontal).

**Fig. 3:**
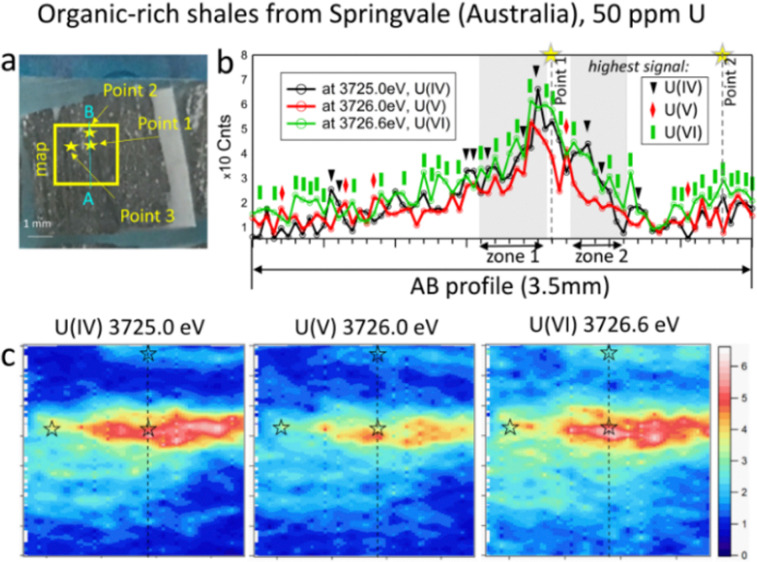
(a) Photo of Springvale organic-rich shale (Australia) containing 50 ppm of U, with the selected map area of 3.5 mm (vertical) × 4.0 mm (horizontal); (b) intensity profiles along the AB line; and (c) HERFD-XRF maps showing the distribution of U(iv), U(v), and U(vi) in the selected area.

The beam size during HERFD-XRF measurements was 50 microns vertically and 2 mm horizontally, suitable for bulk-rock analyses. A relatively large area of the sample (millimetres in size) can be measured in a short time. The time required for each 3 mm by 5 mm map in size (Fig. 2b, 126 points, 10.5 s per point) was 22 minutes. For samples with higher U concentrations, the counting time can be reduced to 4 min for a map of this size (126 points, 1 s per point). To obtain higher-quality HERFD-XRF images, the oversampling technique can also be applied (Fig. SI2, ESI†). Using a step size smaller than the beam size enhances image resolution without the need for additional image processing. However, acquisition time increases significantly, *i.e.* 76 min instead of 22 min (Fig. SI2, ESI†). Applying interpolation to HERFD-XRF images can improve their aesthetic quality (Fig. 2b and 3c) while maintaining the original resolution.

By comparing the HERFD-XRF maps measured in the phosphorite grainstone sample (Fig. 2), we can conclude that each oxidation state displays a unique distribution, with the maximum signal intensities appearing in different regions. This variation may arise from the competitive behaviour among the oxidation states, where the intensity increase in one state is associated with an intensity decrease in another oxidation state. The complex distribution may result from different factors, such as varying kinetics associated with the fixation of specific U species or distinct sources of U.

In contrast to the phosphorite grainstone sample from USA, U(iv), U(v), and U(vi) are broadly collocated in the organic-rich shales from Springvale, Australia (Fig. 3). However, a more detailed analysis of the maps reveals significant differences. Notably, the area with a near-zero signal at 3726.6 eV is smaller compared to the other oxidation states, indicating that U(vi) is the most abundant state in this sample (see blue areas in Fig. 3c). Further confirmation is provided by the A–B intensity profile (Fig. 3). Among 72 points in this profile (Fig. 3b), the signal for U(vi) dominates in 38 points (green rectangles), while U(iv) – in 10 points (black triangles) and U(v) – in 5 points (red diamonds in Fig. 3b). The remaining 19 points exhibit nearly equal signals across the three oxidation states.

To determine the ratio of U oxidation states present in the Springvale shale, HERFD-XANES were measured at selected points (indicated by stars in Fig. 3) and were analyzed with the ITFA software package^36^ (*cf.* ESI,† and Fig. 4). This analysis indicates that 35–70% of U(vi), 30–41% of U(iv), and 0–23% of U(v) are present in different regions of the sample. For the analysis, UO_2_,^25^ UMoO_5_^29^ and β-UO_3_^27^ were used as pure U(iv), U(v) and U(vi) references, respectively. Consistent with the HERFD-XRF maps measured independently (see intensity profiles in Fig. 3b), the HERFD-XANES results confirm that U(v) is the least abundant oxidation state. The modelled fraction of U(v) (∼20 to 23%) is above the analysis limit, estimated at ∼10%, for spectra exhibiting mixed U(iv), U(v), and U(vi) oxidation states. Even higher amounts of U(v) were observed in the HERFD-XANES spectra recorded for the phosphorite grainstone sample from the USA (>45% of U(v), *cf.* Fig. SI5, ESI†). Thus, our data reveal the significant presence of U(v) in such sedimentary rocks.

**Fig. 4:**
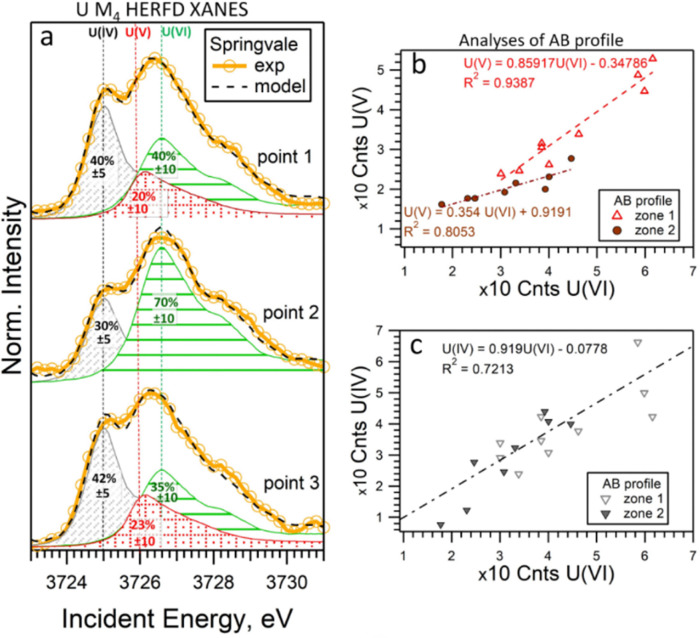
(a) U M_4_ edge HERFD-XANES spectra measured at individual points in Springvale organic-rich shale (see Fig. 3), along with quantitative models and component contributions. The lines correspond to 3725.0 eV, 3726.0 eV, and 3726.6 eV. (b) and (c) Correlations of intensities across two regions (zone 1 and 2) of the AB profile shown in Fig. 3b. For this analysis, UO_2_, UMoO_5_ and β-UO_3_ were used as pure U(iv), U(v) and U(vi) references, respectively.

It should be noted that the choice of U references may impact the HERFD-XRF data and ITFA analysis. Our experience with HERFD-XANES data collection shows that the position and shape of the HERFD-XANES spectra recorded on pure U(iv) and U(v) compounds exhibit only slight variations (*cf.* ref. 24 where we report data on UO_2_ in comparison with UCl_4_, as well as HERFD-XANES data recorded on NaUO_3_ and KUO_3_). However, the shape of the U(vi) HERFD-XANES spectrum can vary depending on the local structure.^24,37^ To assess the sensitivity of the U(vi) spectral shape reference to the local structure and its impact on the analysis, we conducted an additional evaluation using U(vi) as UO_2_(NO_3_)_2_·6H_2_O (*cf.* Fig. SI3 and SI4, ESI†). The best agreement between the experimental data and the fit was achieved using the model with β-UO_3_ reference. Fig. SI3 (ESI†) shows that the HERFD-XANES spectrum recorded for the Springvale sample (point 2) exhibits a characteristic feature at ∼3732 eV, similar to UO_2_(NO_3_)_2_·6H_2_O. At the same time, the feature at ∼3728 eV corresponds to that observed for β-UO_3_. This suggests the potential of HERFD-XANES for U(vi) speciation; however, confirming the specific phase would require additional analytical techniques.

For further insight into the special distribution of U(iv), U(v), and U(vi) in organic-rich shales, we conducted additional analyses along a transect (the AB profile from the HERFD-XRF maps, Fig. 3). Fig. 4b illustrates the intensity of U(v) plotted against the intensity of U(vi) for two regions of the AB profile (zones 1 and 2). In these regions, two distinct linear correlations are observed, indicating variations in U(v)/U(vi) in addition to changes in U concentration. In contrast, the correlation between U(iv) and U(vi) shows a single trend (Fig. 4c), suggesting a constant U(iv)/U(vi) ratio across all points, with variations primarily attributed to changes in the total U concentration (*cf.* Fig. SI6, ESI†). It is important to note that this organic-rich shale originated from marine sediments, with U(vi) from seawater acting as the primary source of U. This context allows us to interpret the correlations in Fig. 4 as suggesting similar incorporation pathways for U(iv) and U(vi), while U(v) might be more sensitive to other processes and conditions (*e.g.*, mineral interactions or early diagenetic remineralisation).

Identifying the U behaviour in these rocks poses significant challenges, as the mineral grain sizes are often very small (down to the nanoscale) and are accompanied by a complex composition of organic matter, which plays a crucial role in U geochemistry.^38^ Consequently, the correlations among the U oxidation states observed over large areas in the rocks (*i.e.*, at the millimetre scale) suggest that sorption and reduction are closely linked processes in U behaviour in such environments. Additionally, the correlation of U distribution with other elements (such as S, C, P, and Fe) may enhance our understanding of these processes, particularly regarding U(v).

Our novel findings demonstrate that U immobilization can occur without the complete reduction of U(vi) in anoxic sediments. This aligns with previous data from the water column of the anoxic Black Sea;^39^ experiments on U(VI) sorption by organic matter;^40^ and earlier reports on the redox state of uranium in organic-rich continental sediments.^41^ The presence of U(vi) in these ancient sedimentary rocks, dated to ∼260 million years for the phosphorite and ∼100 million years for the Australian organic-rich shales, indicates that U(vi) mobility can be limited under such geological settings. We demonstrate that the less soluble U(iv) species are the final product of reduction processes, likely resulting from both biogenic and abiogenic origins.^12^ Our current knowledge of U(v) geochemistry is incomplete. Among more than 250 U minerals,^42^ only a few are known to contain U(v), for example wyartite.^4^ However, this particular mineral is unlikely to be present in organic-rich shales because it is known to form *via* the alteration of high-grade uranium ores under oxidizing conditions. Therefore, other possible forms of U(v) should be considered. Urananite, also known as pitchblende, is one of the most abundant uranium minerals, with a general chemical composition of UO_2+*x*_. This composition can include pure U(iv) oxide (UO_2_) as well as U_4_O_9_ or even U_3_O_7_ and U_3_O_8_. All these UO_2+*x*_ contain significant fractions of U(V), *i.e.* 50% in U_4_O_9_ and 66% in U_3_O_7_ and U_3_O_8_.^25,27^ The possibility of the formation and long-term preservation of U(v)-rich UO_2+*x*_ phases in sedimentary processes under highly reduced conditions requires further investigations. The other possible states of U(v) can be related to organic matter and biological activity. Some bacteria are known to be able to reduce U(vi) and stabilize U(v).^5,43^ The different correlations found in organic-rich shales may originate from kinetic rates of reduction and precipitation during sediment formation under anoxic conditions^32,43,44^ through a combination of biotic and abiotic processes.^12^ Generally, U(v) has been shown to be stable and significant primarily through experimental studies.^29,32,43–46^ Our results further demonstrate that U(v) accounts for a significant fraction of the total uranium content and that U speciation is heterogeneous at the μm-scale in these sedimentary systems.

Overall, we have demonstrated that HERFD-XRF imaging at the U M_4_ edge is a powerful technique for visualizing the distribution of uranium oxidation states in complex, heterogenous natural samples. Significant fractions of U(v) were identified and quantified at the rock level for the first time, revising the current understanding of U geochemistry in sedimentary processes, which had previously overlooked the role of U(v). The exceptional data obtained from this technique hold substantial potential for applications across various fields, including geochemistry, biology, metallurgy, environmental sciences and nuclear material sciences.

This research was funded by the European Research Council (ERC) under grant agreement No. 759696. K. L, A. C., and F. M. acknowledge the support by NSF-EAR Grants 2322205 and 2322206. The authors would like to thank K. Guerin and A. Burnham for their insightful discussions and assistance with the manuscript preparation. E. B. and C. J. B. publish with the permission of the CEO of Geoscience Australia.

## Data availability

Data for this article are available at https://doi.org/10.15151/esrf-dc-2017287772.

## Conflicts of interest

There are no conflicts to declare.

## Supplementary Material

CC-061-D4CC06211F-s001
