# Supplementary material for: Advanced 2D XRF imaging of uranium oxidation states using HERFD at the U M4 edge
Source: Chem Commun (Camb). 2025 Feb 5;61(19):3840–3. doi: 10.1039/d4cc06211f (PMC11816045; doi:10.1039/d4cc06211f)
Supplement: CC-061-D4CC06211F-s001 [file CC-061-D4CC06211F-s001.pdf]

Supplementary Information (SI) for Chemical Communications.
This journal is © The Royal Society of Chemistry 2025

Supplementary Information

Advanced 2D XRF imaging of uranium oxidation states using HERFD at the U M_4 edge

Elena F. Bazarkina[a,b], Kimberly V. Lau[c], Anthony Chappaz[d], Evgeny Bastrakov[e], Barbara Etschmann[f], Joël Brugger[f], Madeline Marshall[g], Frances M. Meyer[c], Christopher J. Boreham[e], Lucia Amidani[a,b] and Kristina O. Kvashnina[a,b]

[a] Institute of Resource Ecology, Helmholtz Zentrum Dresden-Rossendorf (HZDR), PO Box 510119, 01314 Dresden, Germany.
[b] The Rossendorf Beamline at ESRF, The European Synchrotron, CS40220, 38043 Grenoble Cedex 9, France.
[c] Dept of Geosciences and the Earth and Environmental Systems Institute, Penn State University, University Park PA 16802
[d] STARLAB, Department of Earth and Atmospheric Sciences, Central Michigan University, 17 MI 48859, USA.
[e] Geoscience Australia, GPO Box 378, Canberra, ACT 2601, Australia.
[f] School of Earth Atmosphere and Environment, Monash University, Clayton, VIC 3800, Australia
[g] Department of Earth and Environment, Albion College, Albion MI 49224
† Supplementary Information available: details of samples, method and analysis. See DOI: 10.1039/x0xx00000x

I. *Description of samples*

Two different types of sedimentary rocks were selected: phosphorite grainstone from USA and organic-rich carbonaceous shales (also called black shales) from Australia.

1. *Phosphorite grainstone (USA)*

Previous work characterizing the U oxidation state in geologic and modern apatite using chemical separation methods identified U(IV) as the initial U valence state, with U(VI) present due to later oxidation (Clarke et al., 1958; Kolodny et al., 2017). This study analysed an apatite-rich sedimentary rock (phosphorite) that was not characterized in prior work. The sample is a peloidal phosphorite grainstone with conodont element fragments from the Permian Phosphoria Rock Complex and was collected from the South Deer Creek stratigraphic section in southeast Idaho (Marshall, 2018). The stratigraphic section was located on an active mine face and therefore surface oxidation was minimal. The age of the Phosphoria Rock Complex has been constrained as 260.57 ±0.07 / 0.14 / 0.31 Ma, i.e. latest Capitanian, Guadalupian ($^{206}Pb/^{238}U$ dating, Davydov et al., 2018). The sample is from the organic- and phosphate-rich Meade Peak Member, with typical organic carbon and P contents of 1.8 wt% and 7.1%, respectively, at this site. The Meade Peak Member contains siliceous, carbonaceous, and calcareous shales that were deposited in a shallow epicontinental sea along the western margin of the ancient supercontinent Pangea. Phosphogenesis is attributed to vigorous upwelling that transported nutrients from the open ocean, leading to high primary productivity in the water column and low dissolved oxygen in bottom waters (Piper and Link, 2002). The depositional P-bearing minerals are predominantly carbonate fluorapatite, which can contain 10^2 ppm levels of U (Clarke et al., 1958; Altschuler, 1980). The U contents in the Meade Peak Member are high, owing to its inferred low-oxygen setting and the prevalence of carbonate fluorapatite (Gulbrandsen, 1966). Whole-rock U concentrations of the Meade Peak Member in this stratigraphic section range from 2.6 to 131 ppm, and U concentrations for the analyzed sample are 79.7 ppm, with total organic carbon and P contents 2.3 and 12.0 wt%, respectively. XRF maps of U show heterogeneous distribution throughout the sample, with highest U concentrations thought to be associated with organic material (Meyer et al., 2024). Authigenic apatite grains have a lower concentration of U, with enriched U in the grain

boundary. This heterogeneity suggests multiple active U burial pathways during deposition of the Phosphoria Rock Complex (Meyer et al., 2024).

The sample was prepared as a polished, 30 µm-thick thin section mounted with epoxy on a 27 x 46 mm glass slide.

2. Organic-rich carbonaceous shales (Australia)

There is no previous work directly characterizing the U oxidation state in ancient and modern organic-rich marine sediments. This study is the first step to cover this knowledge gap. To this end, we selected two samples that come from an organic-rich marine carbonaceous shale and a coquinite - from the mid-Cretaceous Toolebuc Formation of the Eromanga sedimentary basin, Australia (Boreham and Hoffmann, 2012). The age of the Toolebuc Formation is constrained by biostratigraphy as Late Albian to Early Cenomanian (~101 to 98 Ma). This marine formation is one of Australia's most organic-rich sediments. It typically comprises interlayers of carbonaceous shale and coquina (Hoffmann, 1985), with carbonaceous shale layers containing up to 35 weight percent of the total organic carbon (TOC) (Riley and Saxby, 1982). The organic-rich shale layers were deposited under anoxic seafloor conditions (Boreham and Powell, 1987). There is a strong positive correlation between uranium content and TOC in carbonaceous shale (Bastrakov et al., 2023), suggesting a genetic link between U and organic matter. For samples with TOC concentrations between 1 and 11 wt%, U concentrations range from 3 to 80 ppm. Coquinite samples deviate from the observed U-TOC correlation, showing elevated U concentrations, with the highest one reaching 300 ppm. Some of the coquinite samples exhibit an increased phosphorus content. In these, elevated U might be attributed to U in apatite grains associated with skeletal fish remains (Ozimic, 1986; Patterson et al., 1986). These observations suggest a multi-phase (mineral and organic) sink for U (Boreham and Hoffmann, 2012). Uranium is unevenly distributed at the millimetre to nanometre scale (Bastrakov et al., 2023). Nanoscale secondary ion mass spectrometry (NanoSIMS) reveals different associations that often coexist in single samples; nano-particulate U is associated with organic matter matrix or sulphide minerals, whereas phosphate minerals display diffuse U enrichment. Unpublished results of high-energy resolution fluorescence detection (HERFD) X-ray absorption spectroscopy at U L_3 edge (Bastrakov et al., 2023) suggested that a significant proportion of U (~20 to 30%) in carbonaceous shales exists as U(VI), despite the extremely reducing (anoxic to euxinic) conditions during sediment precipitation and diagenesis. Coquinite samples have a higher proportion of U(VI) consistent with the dysoxic depositional environment.

A sample of marine carbonaceous shale (Geoscience Australia sample number 2000052) and a sample of coquinite (Geoscience Australia sample number 1312752) were selected from drillholes BMR Springvale 6 and Westmoreland 2 at depths of 50.95 m and 218.19 m, respectively. Supergene alteration and surface oxidation were minimal. The BMR Springvale 6 sample has a U concentration of 44.26 ppm, with TOC and P_2O_5 contents at 5.89 wt% and 0.49 wt%. The Westmoreland 2 sample shows a U concentration of 297.3 ppm, with TOC and P_2O_5 contents at 2.37 wt% and 5.50 wt%.

The samples were prepared as non-polished, approximately centimeter-sized, 0.5 cm-thick rock slabs mounted with epoxy on a 30 x 50 mm glass slide.

II. Data Acquisition

The measurements were performed at the BM20 ROBL beamline (Scheinost et al.; 2021) at the European Synchrotron Radiation Facility (ESRF, Grenoble, France). The storage ring was operated in the multi-bunch filling mode at 6 GeV with a 200 mA current. The incident energy was selected using

a Si(111) double-crystal monochromator. Two Si mirrors before and after the monochromator were used to collimate the beam and reject higher harmonics. Beam size was estimated to be ~50 µm (vertically) by ~2 mm (horizontally). For all measurements, the sample with the flat surface was placed vertically and rotated by 45° to the incident beam. The incident energy was calibrated using a HERFD XANES of UO_2 at ambient T-P conditions; its M_4 edge XANES maximum energy position was set at 3725 eV. The HERFD measurements were performed using a Johann-type X-ray emission spectrometer in a vertical Rowland geometry. The spectrometer has five Si(220) crystal analyzers with a 1 m bending radius oriented by 75°Bragg angle, and a silicon drift X-ray detector (©Ketek). A helium-filled bag with thin plastic windows was used to fill the optical path sample-crystal analyzers-detector to reduce the absorption of the U Mβ fluorescence signal by the air. The spectrometer was aligned using UO_2 powder excited by the incident X-ray beam at fixed energy above the X-ray absorption edge, i.e. in the non-resonant emission mode (at incident energy 3775 eV).

XRF-HERFD images at the U M4 edge were obtained by measuring the intensity of emitted photons with an energy of 3337.0 eV as a function of the incident energy while scanning the sample. The data points were interpolated. Three maps were recorded at three different incident energies (3725.0, 3726.0, and 3726.6 eV). The units for the HERFD-XRF maps are arbitrary, representing counts from electronics sensitive to X-ray photons.

Table S1. *Details on U M_4 HERFD-XRF image measurements*

Sample	Incident E range (eV)	E step (eV)	Time per point (sec)		Number of HERFD-XANES spectra	Total time (min)
SDC (USA)	3725.0	3.0 x 5.0 (0.1x1)	126	10	22	Fig. 2b
	3726.0	3.0 x 5.0 (0.1x1)	126	10	22	Fig. 2b
	3726.6	3.0 x 5.0 (0.1x1)	126	10	22	Fig. 2b
Springvale	3725.0	3.5 x4.0 (0.05x0.25)	1207	13	268	Fig. 4
	3726.0	3.5 x4.0 (0.05x0.25)	1207	13	268	Fig. 4
	3726.6	3.5 x4.0 (0.05x0.25)	1207	13	268	Fig. 4
Westmoreland	3725.0	1.0 x 5.0 (0.05x 1)	126	10	22	Fig 3a
Westmoreland	3725.0	1.0 x 5.0 (0.05x 0.25)	441	10	76	Fig. 3b

HERFD-XANES spectra were recorded with a 0.2 eV step and a counting time of 5–20 seconds per point (see Table SI2). Multiple HERFD-XANES spectra were collected to improve the signal-to-noise ratio. As a result, the total acquisition time for the final HERFD-XANES spectra ranged from 63 to 168 minutes.

Table SI2. *Details on HERFD-XANES spectra measurements*

Sample	Incident E range, eV	E step, eV	Time per point (sec)	Nb of HERFD-XANES spectra	Total time (min)
SDC (USA) all points	3721-3736	0.2	5	13	104
Springvale					
point1	3721-3736	0.2	15	6	126
point2	3721-3736	0.2	20	6	168
point3	3721-3736	0.2	15	3	63

III. Tests for Potential X-Ray Beam-Induced Damage:

Beam-induced damage was assessed both in reference compounds and the samples. For the reference compounds, two approaches were used: (1) Measuring X-ray intensity at 3725.0 eV, 3726.0 eV, and 3726.6 eV on fresh spots as a function of time (with 0.1sec time counting) over a 5-minute period. In the event of beam damage, the timescan profile would change. Since no such changes were observed, this confirmed the absence of beam-induced alterations. (2) Performing repetitive fast XANES scans (0.1 seconds per point, with a 0.3 eV step in the 3723–3730 eV region) on previously unanalyzed spots. In the event of beam damage, spectra would exhibit variations over time, such as energy shifts and changes in peak intensities. These scans showed no spectral evolution over the 5-minute period, further indicating stability.

For natural rock samples with low uranium concentrations, such as the organic-rich shale and phosphorite, these timescan tests (indicated as 1) above) were unsuitable due to the low HERFD signal. Instead, multiple spectra were carefully compared before averaging. No variations were observed, confirming sample stability under X-ray exposure. It should be noted, that the X-ray beam at the ROBL beamline has a relatively moderate flux and has a horizontally large profile, which minimizes both heat load and potential X-ray-induced chemical changes. Beam-induced damage at ROBL is significantly less pronounced compared to other beamlines with focused beams and higher flux. All tests confirmed the absence of beam-induced damage, validating the stability of uranium in mixed oxidation states (U(IV), U(V), and U(VI)) within the analyzed samples. An example of these tests is shown in Fig. SI1.

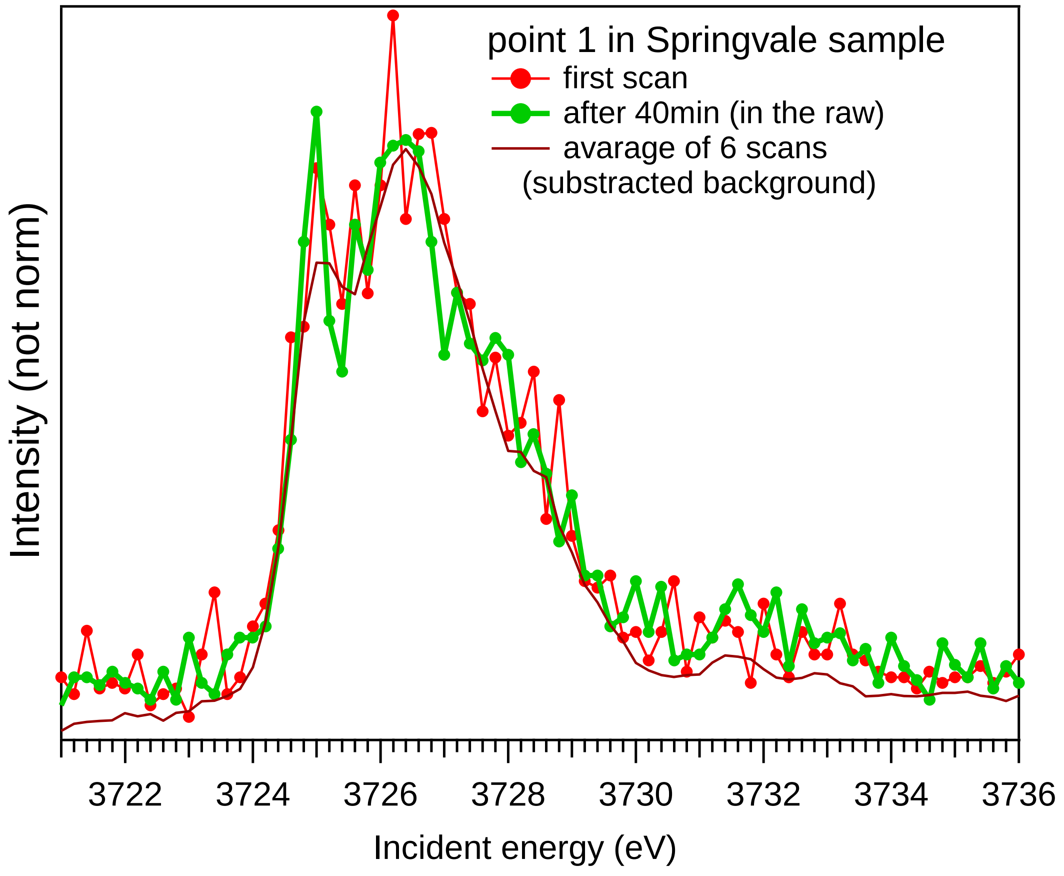

Fig. SI1. *The beam-damage test at point 1 compares the first individual HERFD XANES scan with the last scan in the series and the final averaged spectrum. The absence of energy shifts over time and the reproducibility of the spectra indicate that no beam-induced chemical changes occurred in the sample.*

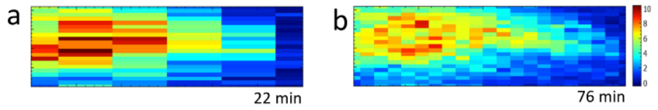

Fig. SI2. *Horizontal oversampling technique for U M_4 edge HERFD-XRF map at 3725.0 eV in 1mm x 5mm area of Westmoreland organic-rich shale (Australia) containing 300 ppm of U. Vertical step is 0.05 mm for both images. Horizontal step and total acquisition time are 1mm and 22min in (a) and 0.25mm and 76min in (b). The beam size is 0.05 x 2 mm (V x H).*

III. Data Processing and Analyses

Data Processing for HERFD-XANES

Individual XANES spectra were initially compared using the PyMca software (Sole et al., 2007). After confirming the stability of the spectral shape (with no observed changes between spectra collected at the same spot), the spectra were averaged. The background was subtracted using a Savitzky-Golay filter with a width of 1, an interpolating polynomial degree of 1, and a derivative order of 0 (see Figure SI1). Following this, the spectra were normalized to the area in the energy range of 3721.0–3726.0 eV.

ITFA Analysis and Linear Combination Fitting (LCF)

The quantification of U(IV), U(V), and U(VI) fractions was performed using Iterative Target Factor Analysis (ITFA) (Rosseberg et al. (2003)). ITFA is a multivariate analysis technique used in spectroscopy, including XANES and XRF, to resolve overlapping signals and extract meaningful chemical components from complex spectral datasets. Principal components analysis (PCA) was used to determine the number independent components that reproduced the major variance of the entire data set. These components were then extracted from the dataset by ITFA, and linear combination fitting (LCF) was used to determine the relative contribution of the independent components to each spectrum. This aanalysis typically is done in two steps:

1. Eigenanalysis: In the first step, eigenanalysis—a linear algebra technique for studying linear transformations—was used to determine the number of linearly independent spectral components. This was done by evaluating eigenvalues and using a semi-empirical indicator function. The number of primary factors was identified at the minimum value of the indicator function.
2. Iterative Target Testing (ITT): In the second step, ITT was applied to the spectra of samples and reference compounds of pure U(IV), U(V), and U(VI), with the number of pure components fixed to three. The ITT process used concentration test vectors for non-orthogonal rotation and independently determined factor concentrations. The final model was an LCF of the independent component spectra identified by ITT.

Choice of Reference Compounds for ITT and LCF

The oxidation states of U(IV), U(V), and U(VI) in the samples were initially unknown. Additionally, the measured spectra in the samples displayed varying mixtures of oxidation states. The mineral sizes in the samples were far smaller than the beam size, and the mineral composition was highly complex. The final ITT and LCF analysis results will always depend on the selection of the references. To account for uncertainties related to unknown pure components in the mixtures, ITT analysis was performed with various sets of standards. Higher uncertainty was observed for U(V) and U(VI) (estimated at 10%) compared to U(IV) (estimated at 5%). Comparisons of the standards and models are presented in the accompanying figures and tables below.

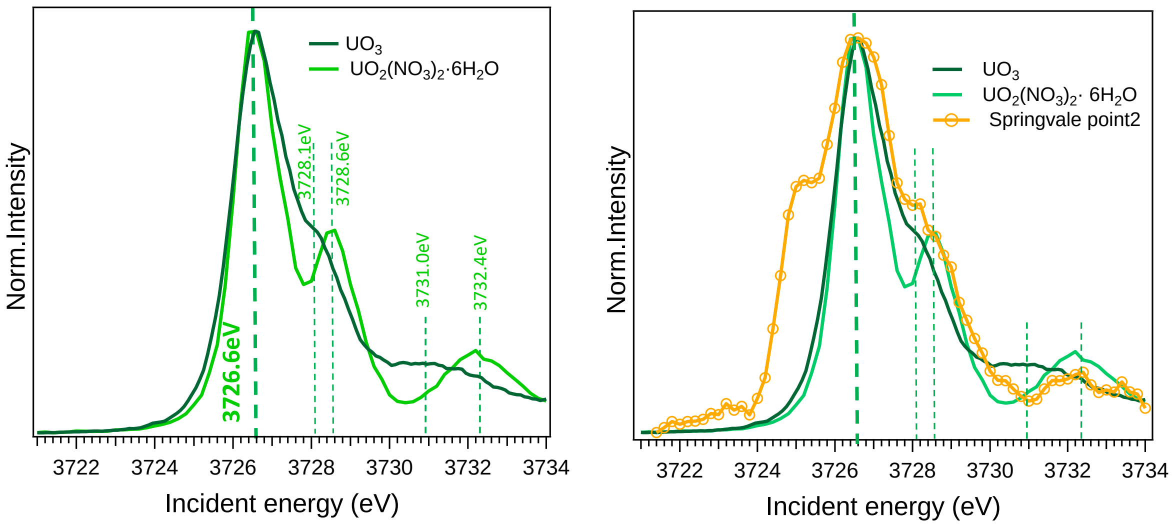

Fig. SI3. *U M_4 edge HERFD-XANES spectra of two types of U(VI) compounds: UO_3 and $UO_2(NO_3)_2$*$6H_2O$, with various post-edge features indicated by vertical dashed lines. The spectrum of point 2 measured in the Springvale black shale sample is also shown and compared to two U(VI) standards. At this point, the ITFA analysis shows 70% of U(VI) contribution. The post-edge features at ~3728 eV are quite close to the UO_3 standard, while the features at ~3732 eV suggest a possible contribution from a uranyl-like compound.*

Table SI3. *ITT analysis of multiple oxidations states in U inorganic-rich shales from Springvale using different references for U(VI):*

	Model with UO_3			Model with $UO_2(NO_3)_2$ *$6H_2O$		
	UO_2 U(IV)	$UMoO_5$ U(V)	UO_3 U(VI)	UO_2 U(IV)	$UMoO_5$ U(V)	$UO_2(NO_3)_2$*$6H_2O$ U(VI)
Point1	40	20	40	41	29	30
Point2	30	0	70	34	10	56
Point3	42	23	35	43	30	27

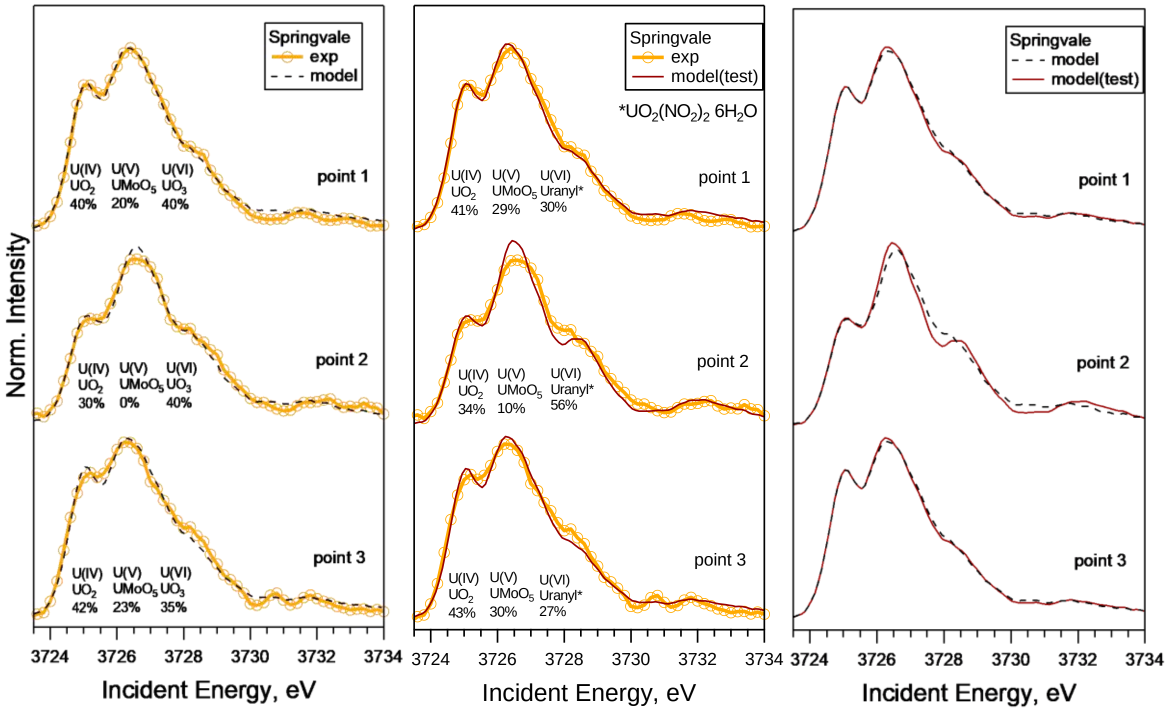

Fig. SI4. *ITFA models with different sets of standards: "model" refers to the configuration using UO_2, $UMoO_5$ and β-UO_3 as references for U(IV), U(V) and U(VI), respectively. The "model(test)" refers to a new configuration using UO_2 , $UMoO_5$ and $UO_2(NO_3)_2 \cdot 6H_2O$ as references for U(IV), U(V) and U(VI). The best agreement between the experimental data and the fit was achieved using the model with β–UO_3 reference.*

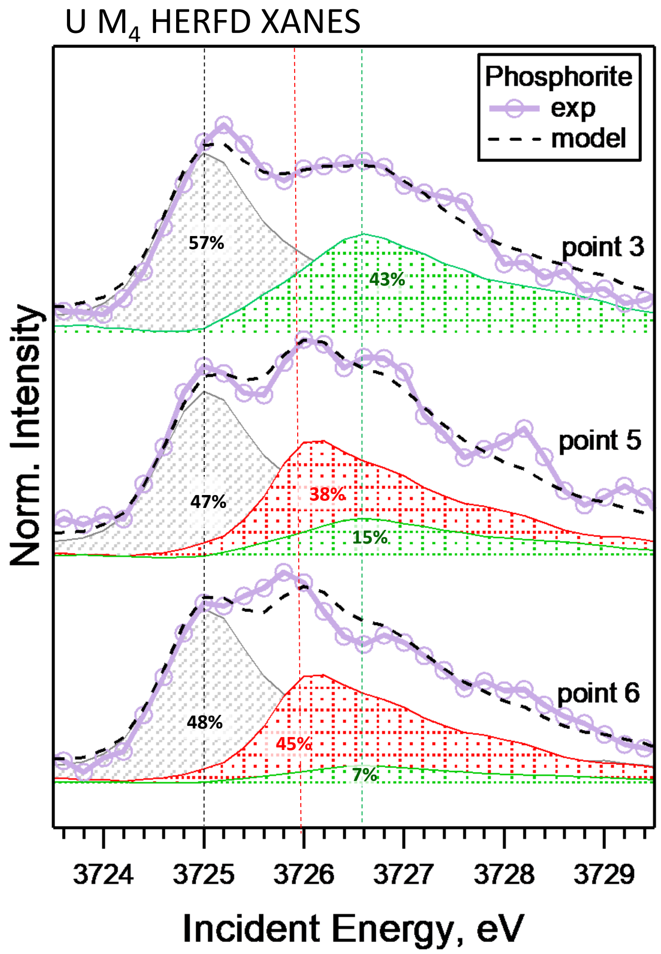

Fig. SI5. *U M_4 edge HERFD-XANES spectra recorded for phosphorite sandstone (USA). The model was created using UO_2, $UMoO_5$, and UO_3 as references for U(IV), U(V), and U(VI), respectively. The models identified up to 45% of U(V).*

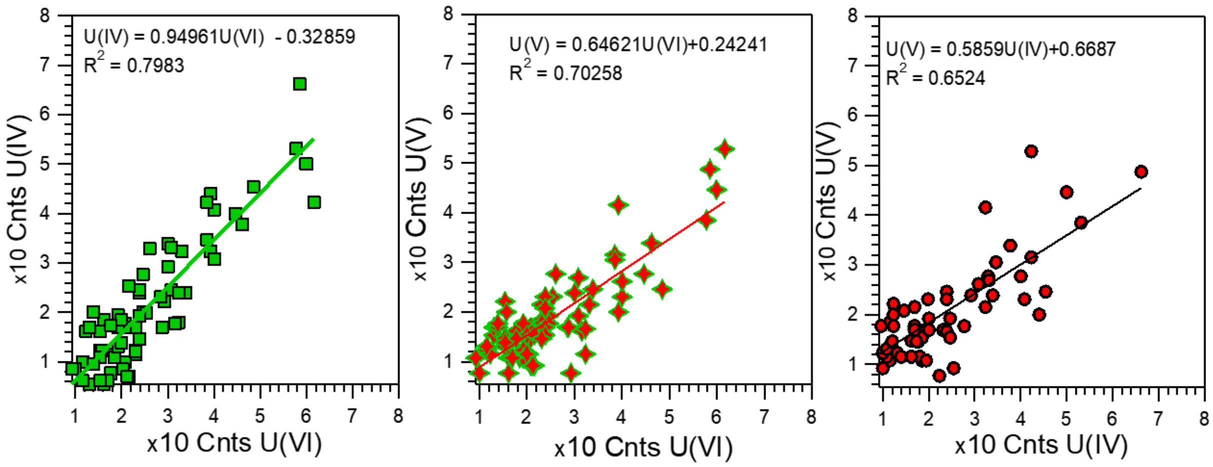

Fig. SI6. *The correlation between non-normalized intensities at 3725.0 U(IV), 3726.0 U(V) and 3726.6eV U(VI) in the A-B intensity profile in the black shale from Springvale (Australia) presented in the Fig. 3b (main text). The best correlation is for U(IV) and U(VI) (R^2=0.7983), and the worst correlation is for U(V) and U(VI) (R^2=0.6524). For some regions of this profile, U(V) and U(VI) show different correlations (see Fig. 5, main text).*

References

Altschuler, Z.S., 1980. The Geochemistry of Trace Elements in Marine Phosphorites Part I. Characteristic Abundances and Enrichment. SEPM Special Publication, Marine Phosphorites Geochemistry, Occurrence, Gen 29, 19–30. https://doi.org/10.2110/pec.80.29

Bastrakov, E., Boreham, C., Edwards, D., Jarrett, A., 2018. Uranium in organic-rich shales as a tool for predicting hydrocarbon potential. 20th Australian Organic Geochemistry Conference. Canberra. 2pp.

Bastrakov, E., Brugger, J., Etschmann, B., Bazarkina, E., Kvashnina, K., Proux, O., Testemale, D., Boreham, C., VanDerWielen, S., Guagliardo, P., 2023. Characterisation of uranium redox state in organic-rich marine sediments of the Cretaceous Toolebuc Formation, Australia, Goldschmidt2023 abstracts.

Boreham, C., Hoffmann, K., 2012. Vertical geochemical profiling of the marine Toolebuc Formation, Eromanga Basin - implications for shale gas/oil potential, Eastern Australasian Basins Symposium IV. Brisbane, QLD, pp. 53-59.

Boreham, C.J., Powell, T.G., 1987. Sources and preservation of organic matter in the Cretaceous Toolebuc Formation, eastern Australia. Organic Geochemistry 11(6), 433-449. doi.org/10.1016/0146-6380(87)90001-5.

Clarke, R.S., Altschuler, Z.S., 1958. Determination of the oxidation state of uranium in apatite and phosphorite deposits. Geochimica et Cosmochimica Acta 13, 127–142.

Cumberland, S.A., Etschmann, B., Brugger, J., Duglas, G., Evans, K., Fisher, L., Kappen, P., Moreau, J.W., 2018. Characterization of uranium redox state in organic-rich Eocene sediments. Chemosphere 194, 602-613.

Davydov, V.I., Crowley, J.L., Schmitz, M.D., Snyder, W.S., 2018. New U–Pb constraints identify the end-Guadalupian and possibly end-Lopingian extinction events conceivably preserved in the passive margin of North America: implication for regional tectonics. Geological Magazine 155, 119–131. https://doi.org/10.1017/S0016756816000959

Gulbrandsen, R.A., 1966. Chemical composition of phosphorites of the Phosphoria Formation. Geochimica et Cosmochimica Acta 30, 769–778. https://doi.org/10.1016/0016-7037(66)90131-1

Hoffmann, K.L., 1985. A palaeoenvironmental study of the northern reaches of the Toolebuc Formation. Honours Thesis. James Cook University of North Queensland, p. 152.

Kolodny, Y., Torfstein, A., Weiss-Sarusi, K., Zakon, Y., Halicz, L., 2017. 238U-235U-234U fractionation between tetravalent and hexavalent uranium in seafloor phosphorites. Chemical Geology 451, 1–8. https://doi.org/10.1016/j.chemgeo.2016.12.032

Marshall, M.S., 2018. Sequence Stratigraphy and Taphonomic Expression of Hiatuses in a Highly Condensed, Organic- and Phosphate-rich Record: Paleoenvironmental Analysis and Paleoecology of the Permian Phosphoria Rock Complex, Southeastern Idaho (Ph.D.). University of Chicago, Chicago, Illinois.

Meyer, F.M., Chappaz, A., Kvashnina, K., Bazarkina, E.F., Marshall, M.S., Garber, J.M., Lau, K.V., 2024. Uranium speciation and heterogeneity in marine phosphorites: insight into the uranium paleo-redox proxy. Goldschmidt 2024 abstracts.

Ozimic, S., 1986. The geology and petrophysics of the Toolebuc Formation and its time equivalents, Eromanga and Carpentaria Basins:, in: Gravestock, D.I., Moore, P.S., Pitt, G.M. (Eds.), Contributions to the Geology and Hydrocarbon Potential of the Eromanga Basin. pp. 119–137.

Patterson, J.H., Ramsden, A.R., Dale, L.S., Fardy, J.J., 1986. Geochemistry and mineralogical residences of trace elements in oil shales from Julia Creek, Queensland, Australia. Chemical Geology 55(1-2), 1-16. doi.org/10.1016/0009-2541(86)90123-3.

Riley, K.W., Saxby, J.D., 1982. Association of organic matter and vanadium in oil shale from the Toolebuc Formation of the Eromanga Basin, Australia. Chemical Geology 37(3-4), 265-275. doi.org/10.1016/0009-2541(82)90082-1.

Piper, D.Z., Link, P.K., 2002. An upwelling model for the Phosphoria sea: A Permian, ocean-margin sea in the northwest United States. AAPG Bulletin 86, 1217–1235.

Scheinost, A. C., Claussner, J., Exner, J., Feig, M., Findeisen, S., Hennig, C., Kvashnina, K. O., Naudet, D., Prieur, D., Rossberg, A., Schmidt, M., Qiu, C., Colomp, P., Cohen, C., Dettona, E., Dyadkin, V., Stumpf T., 2021. ROBL-II at ESRF: a synchrotron toolbox for actinide research. Journal of Synchrotron Radiation 28, 333–349.
